# Nicotinic Acid is a Common Regulator of Heat-Sensing TRPV1-4 Ion Channels

**DOI:** 10.1038/srep08906

**Published:** 2015-03-10

**Authors:** Linlin Ma, Bo Hyun Lee, Heather Clifton, Saul Schaefer, Jie Zheng

**Affiliations:** 1 Department of Physiology and Membrane Biology, University of California School of Medicine, Davis, California, USA; 2 Division of Cardiovascular Medicine, University of California School of Medicine, Davis, California, USA; 3 Institute for Molecular Bioscience, University of Queensland, St Lucia, QLD 4072, Australia

## Abstract

Nicotinic acid (NA, a.k.a. vitamin B3 or niacin) can reduce blood cholesterol and low-density lipoproteins whereas increase high-density lipoproteins. However, when NA is used to treat dyslipidemias, it causes a strong side effect of cutaneous vasodilation, commonly called flushing. A recent study showed that NA may cause flushing by lowering activation threshold temperature of the heat-sensitive capsaicin receptor TRPV1 ion channel, leading to its activation at body temperature. The finding calls into question whether NA might also interact with the homologous heat-sensitive TRPV2–4 channels, particularly given that TRPV3 and TRPV4 are abundantly expressed in keratinocytes of the skin where much of the flushing response occurs. We found that NA indeed potentiated TRPV3 while inhibited TRPV2 and TRPV4. Consistent with these gating effects, NA lowered the heat-activation threshold of TRPV3 but elevated that of TRPV4. We further found that activity of TRPV1 was substantially prolonged by extracellular NA, which may further enhance the direct activation effect. Consistent with the broad gating effect on TRPV1–4 channels, evidence from the present study hints that NA may share the same activation pathway as 2-aminoethoxydiphenyl borate (2-APB), a common agonist for these TRPV channels. These findings shed new light on the molecular mechanism underlying NA regulation of TRPV channels.

Nicotinic acid (NA) is a water-soluble small molecule vitamin. It is the precursor for nicotinamide adenine dinucleotide (NAD^+^), a coenzyme involved in the catabolism of fat. NA has been prescribed for over 50 years to lower the serum concentrations of total cholesterol as well as low-/very low-density lipoprotein whereas raises that of high-density lipoprotein^1^,^2^. The beneficial effect is at least in part attributable to activation of hydroxy-carboxylic acid receptor 2 (HCA2) in adipocytes, leading to a drop of intracellular cyclic adenosine monophosphate (cAMP) level and inhibition of lipolysis^3^,^4^,^5^. However, NA treatment has a very unpleasant side effect commonly called flushing, which is characterized by cutaneous vasodilation and symptoms of hot flashes and burning sensation^6^,^7^,^8^. Since flushing occurs to 90% of patients taking NA, the clinical application has been significantly limited. Indeed, about 1/3 of patients given NA have opted to stop the treatment^7^,^8^. One pathway that mediates the flushing response was thought to be activation of arrestin beta 1 and the downstream effector ERK 1/2 MAP kinase 7 in Langerhans cells and keratinocytes of the skin, leading to release of vasodilatory prostaglandin D2 and E2^9^,^10^,^11^,^12^. Nonetheless, pharmacological blockade of cyclooxygenase (by aspirin) and prostaglandin D2 receptor 1 (by laropiprant) does not fully inhibit flushing^13^,^14^.

In a recent study^15^, we found that NA activates the capsaicin receptor TRPV1, a heat-activated polymodal cellular sensor that mediates the flushing response upon consumption of spicy food^16^,^17^. We observed that NA directly and potently activates TRPV1 from the intracellular side by lowering the activation threshold for heat, causing channel activation at physiological body temperature. In support of the important role TRPV1 plays in NA-induced flushing, we observed that NA-induced increase in blood flow was substantially reduced in *Trpv1^−/−^* knockout mice^15^,^18^. This new finding confirms existing observations that multiple pathways mediate the flushing response^13^,^14^,^19^,^20^, and suggests novel methods for inhibiting flushing to improve patient compliance.

TRPV1 belongs to a group of homologous heat-sensing TRPV channels including TRPV2, TRPV3, and TRPV4 that share substantial structural and functional properties such as involvements in cardiovascular functions^21^,^22^. Like TRPV1, TRPV2–4 channels are heat sensors but exhibit distinct activation threshold temperatures^22^. TRPV1–4 channels also share the common polymodal activation property. In particular, 2-Aminoethoxydiphenyl borate (2-APB) is a common activator for TRPV1-3 and a mutant TRPV4^23^. Our observation of NA-induced TRPV1 activation raises the question whether these TRPV1 homologs can also be targeted by NA. In the present study, we systematically examined the responses of TRPV2–4 to both intracellular and extracellular NA. In addition, we studied the effect of extracellular NA on TRPV1, an important issue given that under clinical settings the extracellular NA concentration is expected to be higher than the intracellular NA concentration.

## Results

### Effects of Extracellular NA on TRPV1 Activation

**Figure 1:**
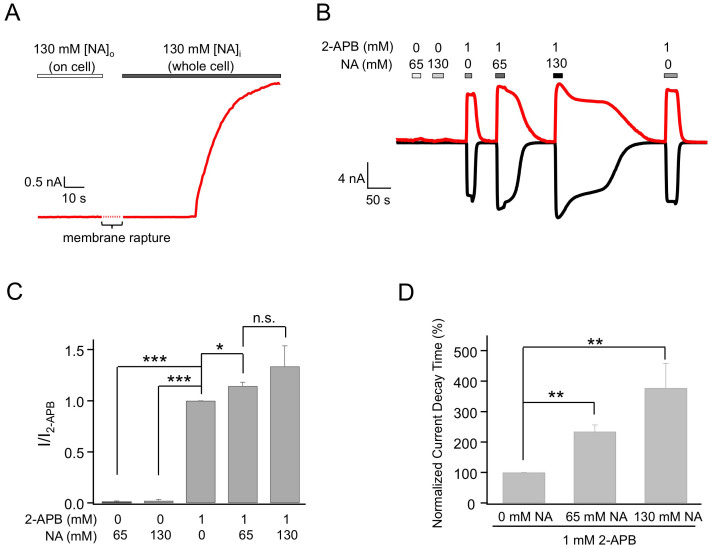
Extracellular nicotinic acid dramatically delays deactivation of 2-APB-induced TRPV1 currents. (A) Representative current trace from an on-cell recording with 130 mM NA in the pipette followed by membrane rapture, leading to whole-cell patch-clamp recording. (B) Representative whole-cell currents induced by 2-APB and NA applied extracellularly, recorded at +80 mV (top trace) and −80 mV (bottom trace). (C) Comparison of current amplitudes. *n* = 3–6. (D) Comparison of the slowdown in the deactivation rate, quantified as the time it took for the current to decline to 50% of the peak level. *n* = 3 to 5. *, p < 0.05; **, p < 0.01; ***, p < 0.005; n.s., not significant.

Our reported study showed that NA directly and strongly activates TRPV1 from the intracellular side^15^. In contrast, no channel activation was observed when NA was applied extracellularly. This can be seen in Figure 1A&B (n = 3, P < 0.005). However, when NA was added extracellularly together with 2-APB, we observed two effects. The efficacy of 2-APB activation was increased in the presence of NA by 14.5 ± 3.6% (n = 3, P < 0.05; Figure 1 B&C, Table 1). More interestingly, the deactivation process of 2-APB-induced activation was remarkably prolonged (Figure 1 B&D). When 2-APB was applied alone, it took 27.0 ± 3.7 s (n = 6) for the current to decrease to one-half of its peak amplitude (time to I_half_). When NA was co-applied with 2-APB, the deactivation time was more than doubled (n = 3, P < 0.01). Increasing NA concentration also extended the deactivation process (Figure 1B&D). The extended channel activity would substantially intensify the flushing response caused by TRPV1 activation. Interestingly, the prolongation effect on deactivation was completely reversible. After channels were completely closed at the end of the elongated deactivation, applying 2-APB alone again evoked currents with normal activation and deactivation kinetics (Figure 1B). Hence, like intracellular NA, extracellular NA also exerts potentiating effects on TRPV1 by both promoting and prolonging channel activation.

### Effects of Intracellular NA on TRPV3

**Figure 2:**
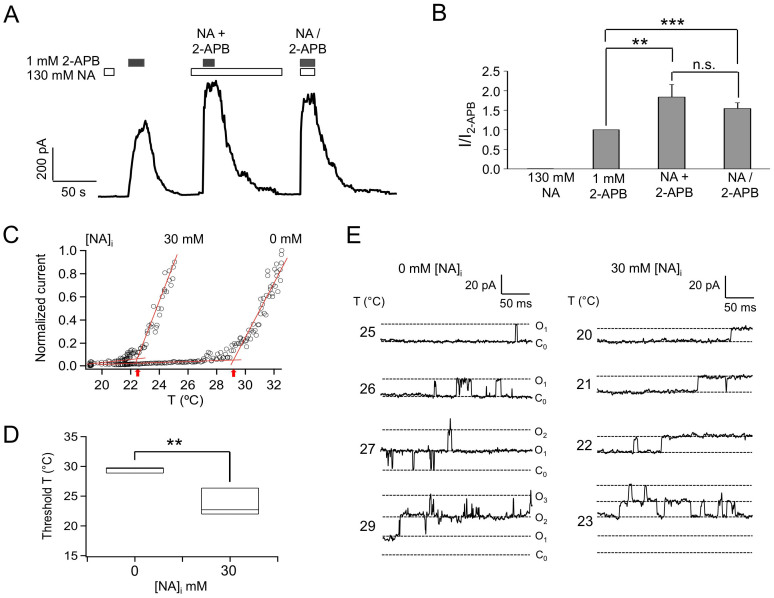
Intracellular NA does not activate TRPV3 but potentiates its activation by 2-APB. (A) Representative current traces from inside-out patches with NA or 2-APB or both applied intracellularly as indicated. (B) Comparison of current amplitudes. *n* = 4. (C) Comparison of heat-induced TRPV3 currents in the absence or presence of 30 mM NA. Dotted lines are fits to the leak current and channel current; arrows indicate the activation threshold temperature. (D) Comparison of threshold temperatures. In this Box-and-whisker plot, the whisker top, box top, line inside the box, box bottom, and whisker bottom represent the maximum, 75^th^ percentile, median, 25^th^ percentile, and minimum value of each pool of conductance measurements, respectively. *n* = 3. **, p < 0.01. (E) Example single-channel traces exhibit heat-dependent activation of the channel in the absence (left) or presence (right) of 30 mM NA. Note that the single-channel current amplitude increased upon temperature increase.

Our previous results suggest that heat activation of TRPV1 at body temperature is likely involved in the flushing response caused by NA^15^. Is TRPV1 the only ion channel target for NA? Particularly, since the heat-sensing TRPV2-4 channels share substantial sequence homology (40–50%) and functional properties with TRPV1, it is important to test whether these channels can also be activated or potentiated by NA. TRPV3 is in particular attractive because it is highly expressed in keratinocytes and it has an even lower heat activation threshold (~30°C) than TRPV1 (~40°C)^22^. As shown in Figure 2A, in inside-out patches from which 1 mM intracellular 2-APB (a saturating concentration for TRPV3) evoked a fairly large TRPV3 current, 130 mM NA caused only marginal channel activation at room temperature. Quantitatively, the efficacy of NA on TRPV3 activation was only 0.6 ± 0.1% (*n* = 5) of that of 2-APB (Figure 2B, Table 2). Nonetheless, when applied together, NA could substantially potentiate the activation of TRPV3 by 2-APB (Figure 2A), increasing the efficacy of 2-APB by 81.2 ± 28.4% (n = 5) (Figure 2B, Table 2). These observations suggest that potentiation of TRPV3 by NA may also contribute to the overall flushing response.

To further examine the effect of NA on TRPV3, we compared heat-induced activation of the channel in inside-out patches in the absence or presence of NA. In the absence of NA, raising the recording temperature to near 30°C started to activate the channel, with the activation threshold temperature quantified to be 29.42 ± 0.28°C (*n* = 3) (Figure 2C&D). We found that including 30 mM NA in the heated solution made it much easier to activate the channel, reducing the threshold temperature substantially to 23.69 ± 1.35°C (*n* = 3). Single-channel recordings further confirmed that as the recording temperature approached the activation threshold temperature, channel activity started to increase (Figure 2E). This finding suggests that NA dynamically regulates the activity of TRPV3, which may contribute to the flushing response.

### Effects of Intracellular NA on TRPV4

**Figure 3:**
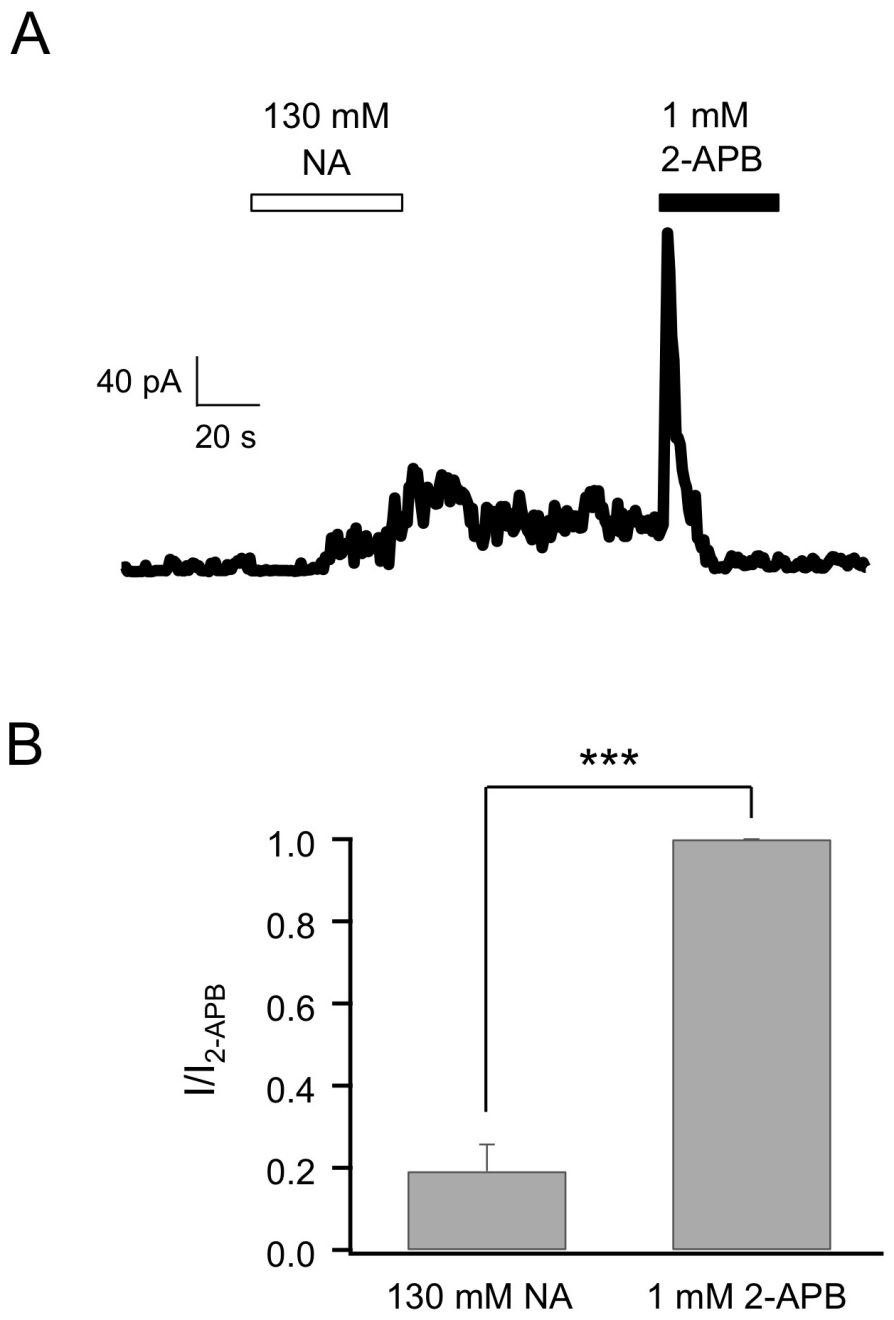
Intracellular NA barely activates TRPV4_dm. (A) Representative current trace of TRPV4_dm from an inside-out patch recording with NA or 2-APB applied intracellularly as indicated. (B) Comparison of the efficacy of 130 mM NA relative to 1 mM 2-APB in activating TRPV4_dm. *n* = 4. ***, p < 0.005.

TRPV4 has similar heat-activation profile as TRPV3, but is insensitive to 2-APB^23^. To facilitate functional study, we used a TRPV4 mutant (N456H/W737R, named TRPV4_dm) which confers 2-APB sensitivity on TRPV4^24^. Similar to the case for TRPV3, intracellular NA activated TRPV4_dm to a very limited extent (Figure 3). Compared to 1 mM 2-APB (a saturating concentration for TRPV4_dm), the efficacy of 130 mM NA was only 19.2 ± 0.1% (n = 4, *P* < 0.005; Figure 3B, Table 2). Therefore, TRPV4 is unlikely to be a major player in NA triggered flushing response. Since 2-APB is not an agonist for the wildtype TRPV4, we did not further characterize the effect of NA on 2-APB induced TRPV4_dm current.

### Effects of Intracellular NA on TRPV2

**Figure 4:**
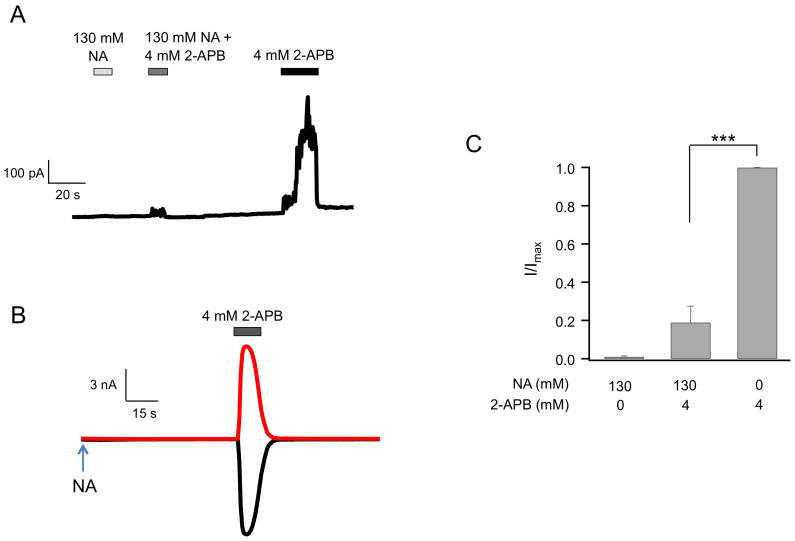
Intracellular NA does not activate TRPV2 but strongly inhibits 2-APB-induced current. (A) A representative TRPV2 current trace from an inside-out patch recording with NA and 2-APB applied on the intracellular side independently or jointly. (B) Representative whole-cell patch-clamp currents of TRPV2 at +80 mV (top) and −80 mV (bottom) with 130 mM NA in the pipette solution and 4 mM 2-APB perfused extracellularly as indicated (*n* = 6). (C) Comparison of TRPV2 currents activated by 130 mM NA with or without 4 mM 2-APB. *n* = 6. ***, p < 0.005.

TRPV2 was completely insensitive to intracellular NA. We tested the effect of intracellular NA on TRPV2 in two ways. First, we conducted inside-out patch recordings with NA perfused into the bath. No activation by NA was observed in TRPV2-expressing cells, though 4 mM extracellular 2-APB (a saturating concentration for TRPV2) induced large currents (Figure 4A). To confirm this observation, we used the whole-cell configuration with NA added to the pipette solution. Again, TRPV2 was completely inert to NA, but could be activated by 2-APB (Figure 4B). Interestingly, we observed that co-application of NA and 2-APB elicited significantly lower currents than 2-APB alone (Figure 4 A&C, Table 2), indicating that NA inhibits 2-APB-induced TRPV2 activation. The fact that NA inhibits 2-APB-induced currents suggests the possibility that the two molecules may compete for common binding sites.

### Effects of extracellular NA on TRPV2–4 channels

**Figure 5:**
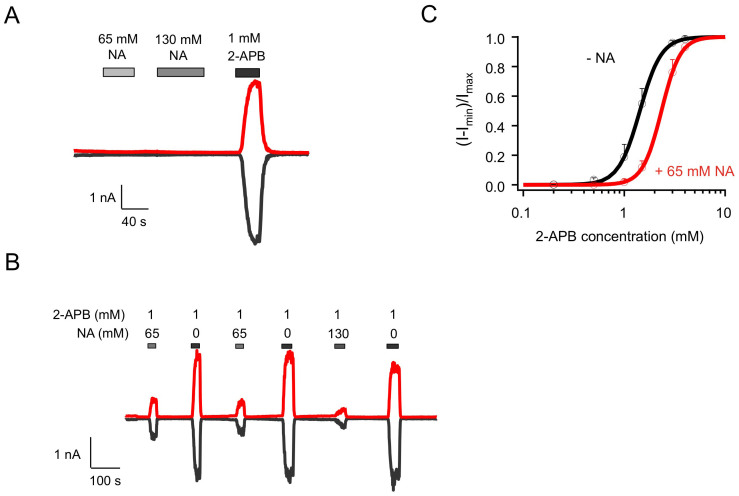
Extracellular NA does not activate TRPV2 but strongly inhibits 2-APB-induced current. (A&B) Representative whole-cell patch-clamp currents of TRPV2 with NA and/or 2-APB applied extracellularly as indicated. (C) 2-APB dose-response curves for TRPV2 without (black) or with (red) 65 mM NA, fitted to a Hill equation with the following parameters (EC_50_ and slope factor): without NA, 1.43 ± 0.04 mM and 4.09 ± 0.5 (*n* = 4); with NA, 2.34 ± 0.17 mM and 4.64 ± 0.98 (*n* = 3).

**Figure 6:**
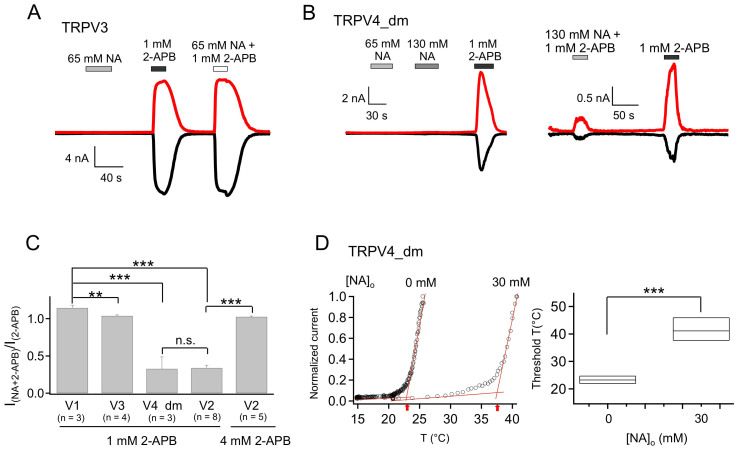
Extracellular NA does not activate TRPV3 or TRPV4_dm but strongly inhibits 2-APB-induced TRPV4_dm current. (A&B) Representative whole-cell current of TRPV3 (A) or TRPV4_dm (B) with NA and/or 2-APB applied extracellularly as indicated. (C) Comparison of the potentiation or inhibition effects of combining extracellular 65 mM NA with 2-APB (concentration as indicated) applied on different TRPV channels. (D) Representative heat-induced TRPV4_dm activation in the absence or presence of 30 mM NA (left) and comparison of the activation threshold temperatures (right). *n* = 3. ***, p < 0.005.

Since intracellular and extracellular NA have different effects on TRPV1, we examined effects of extracellular NA on TRPV2–4 channels with whole-cell patch-clamp recordings. Extracellular NA of high concentrations did not have any observable potentiation effects on TRPV2 (Figure 5A), TRPV3 (Figure 6A) or TRPV4_dm (Figure 6B, left panel). Interestingly, in contrast to a sensitization effect on 2-APB-induced activation of TRPV1, extracellular NA dramatically reduced the activation efficacy of 2-APB on both TRPV2 (Figure 5B) and TRPV4_dm (Figure 6B, right panel), but had no influence on activation of TRPV3 by 2-APB (Figure 6A) (see also Table 1). Consistent with an inhibiting effect, the 2-APB dose-response curve for TRPV2 was right-shifted, with the EC_50_ value determined by a Hill fit (Equation 1) changed from 1.43 ± 0.04 mM (n = 4) without NA to 2.34 ± 0.17 mM (n = 5) with 65 mM NA (Figure 5C). As summarized in Figure 6C and Table 1, compared to 2-APB alone, co-application of extracellular NA potentiated the activation effects of 2-APB on TRPV1 to 114.5 ± 3.6% (n = 3), decreased the activation effects of 2-APB on TRPV2 and TRPV4_dm to 33.9 ± 3.5% (n = 8) and 32.7 ± 16.0% (n = 3) respectively, and had insignificant effects on 2-APB potentiation of TRPV3 (103.8 ± 1.2%, n = 4). It is however interesting to note that, when the concentration of 2-APB was increased to 4 mM, the attenuation effects of extracellular NA on TRPV2 activation was eliminated (I_NA + 2-APB_/I_2-APB_ = 102.5 ± 1.0%, n = 5, *P* < 0.001 compared to 1 mM 2-APB; Figure 6C), again suggesting competition between NA and 2-APB for the same binding sites.

Since extracellular NA inhibited TRPV4, we examined the effect of NA on its heat activation. When 30 mM NA was included in the heated solution, we observed that temperature-dependent activation was substantially shifted to higher temperatures, with the activation threshold temperature elevated from 23.27 ± 0.78°C (in the absence of NA; n = 3) to 41.55 ± 2.38°C (in the presence of NA; n = 3) (Figure 6D). This large gating effect on heat activation is consistent with NA being an inhibitor for TRPV4.

## Discussion

NA treatment is one of the oldest anti-dyslipidemias medications known to reduce the risk of mortality from cardiovascular disease. However, due to the strong and prevalent flushing side effect, its beneficial potentials are far from being fully realized. Identifying the molecular pathways mediating the flushing response and means to reduce or even eliminate it will have important impacts on patient compliance. Indeed, the flushing response (but not the beneficial antidyslipidemic effect) exhibits tachyphylaxis properties, decreasing substantially after continuous treatment^9^,^10^,^11^. This suggests that flushing is mediated by pathway(s) distinct from the antidyslipidemic pathway. Observations from our recent studies^15^,^18^ and the present study suggest that the heat-sensing TRPV channels may both contribute to and regulate the flushing response at physiological body temperature. Our results thus support the notion that, under clinical settings when NA is administrated at dosages much higher than the normal daily intake level, this vitamin molecule hits polymodal heat-sensing TRPV channels as unintended targets to cause the flushing side-effect. Future clinical practice needs to include means to prevent or lessen these unintended actions. These findings thus point to novel potential approaches to reduce the flushing response.

Results from the present study also demonstrate that effects of NA on the homologous TRPV channels are distinct depending on the channel type and which side of the cell membrane NA is applied. Overall, the effects on TRPV1 and TRPV3 are potentiating while those on TRPV2 and TRPV4 are inhibitory. These differences may be the results of distinct structural properties of these ion channels. The atomic structure of TRPV1 has been recently revealed by a cryo-EM study^25^,^26^, while little is known about the detailed structures of TRPV2-4. Revealing the structural and mechanistic bases for these functional differences in future studies may contribute to a better understanding of this important group of cellular heat and nociceptive sensors.

One intriguing observation from the present study is that extracellular NA substantially delays the deactivation of TRPV1 from 2-APB activation. How is this type of gating effects achieved at the molecular level? The phenomenon implies that NA may stabilize the open conformation induced by 2-APB. Through a mutual interaction of allostery, 2-APB would also stabilize the open conformation induced by NA. Multiple extracellular channel structures are involved in the TRPV1 activation gating process. For example, the binding sites for proton and the spider toxin DkTx are located in the pore loop^25^,^26^,^27^,^28^, which is also a critical component of the heat-activation pathway^29^,^30^,^31^,^32^,^33^,^34^. Conformational rearrangement of the pore turret has been observed upon heat activation of TRPV1^30^,^31^. Hypothetically, some of these activation conformational rearrangements may be stabilized by NA, which causes 2-APB to be trapped in its binding sites either directly or again through allosteric coupling, so that it takes a much longer time to come off the channel.

Consistent observations from this study and our previous study point to the possibility that NA may bind to some of the same binding sites of 2-APB to activate TRPV channels. It was previously concluded that, because there is no direct competition between NA and capsaicin or the TRPV1 antagonist capsazepine, it is unlikely that NA binds to the capsaicin-binding sites to activate TRPV1^15^. The presence of gating effects of NA on the capsaicin-insensitive TRPV2–4 channels is also consistent with this view. Our results suggest that NA may occupy with much lower affinity the binding sites for 2-APB, which is also a general activator for this group of TRPV channels. Structural resemblance between NA and 2-APB lends further support for this idea.

In summary, clinical treatment of dyslipidemias patients using NA may affect the activities of heat-sensing TRPV1–4 ion channels. Understanding the potential contribution of these effects to the flushing response and how to lessen or prevent them may lead to improvement of patient compliance in the beneficial and safe NA treatment.

## Methods

### cDNA Constructs and Cell Transfection

Mouse TRPV1–4 cDNAs were inserted into the mammalian expression vector pEYFP-N3. Expression of these constructs yielded channels with an enhanced yellow fluorescence protein (eYFP) tagged to the C-terminus. The fluorescent tag facilitated identification of positively transfected cells and was shown to have undetectable influence on channel functions^35^. The mTRPV4 cDNA contained two point mutations (N456H/W737R) to introduce 2-APB sensitivity to the channel^24^. This TRPV4_dm cDNA construct was kindly provided by KeWei Wang at Peking University.

HEK293_tsA201 cells were cultured in a DMEM medium supplemented with 10% FBS, 2 mM L-glutamine, 1% (v/v) non-essential amino acids, at 37°C with 5% CO_2_. Transient transfection was conducted using Lipofectamine 2000 (Invitrogen, Grand Island, NY) according to the manufacturer's instructions. For whole-cell recordings, cells were plated onto glass coverslips coated with 0.1 mg/ml poly-D-lysine 24-to-48 hours before recording.

### Electrophysiological Recordings

Macroscopic patch clamp recordings were done using a HEKA EPC10 amplifier driven by the PatchMaster software (HEKA, Lambrecht, Germany). All recordings were done at room temperature. For inside-out recordings, patch pipettes were pulled from thin-wall borosilicate glass and fire-polished to a resistance of ~2 MΩ. For whole-cell recordings pipettes were pulled from thick-wall borosilicate glass to 3–5 MΩ. A holding potential of 0 mV was used, from which a 300-ms step to +80 mV followed by a 200-ms step to −80 mV was delivered at 1-s intervals. Currents were low-pass filtered at 2.25 kHz and sampled at 12.5 kHz. For whole-cell recordings the capacity current was minimized by amplifier circuitry, and the series resistance was compensated by 65–80%. Standard symmetrical bath and pipette solutions contained 140 mM NaCl, 0.2 mM EGTA, 10 mM glucose and 15 mM HEPES (pH 7.2). Solution switching was achieved with a rapid solution changer RSC-200 (Bio-Logic Science Instruments, Grenoble, France). For clarity, all inside-out recordings are shown as outward currents at +80 mV, while whole-cell recordings are shown as both outward currents at +80 mV and inward currents at −80 mV.

### Temperature Control

Changes in experimental temperature was carried out by in-line heating, as previously described^29^. Briefly, NA-containing solution was heated with an SHM-828 8-line heater driven by a CL-100 temperature controller (Harvard Apparatus). A BAT-12 Microprobe Thermometer (Physitemp Instruments, Inc.) was placed right next to the patch pipette to ensure accurate monitoring of local temperature. Since TRPV3 activated near room temperature in the presence of NA, in order to measure the activation threshold temperature, we first cooled the bath solution below 20°C by placing the solution reservoir in an ice water baker. The current-temperature relationship had two phases, a slow increase (likely reflected temperature-dependent increase in the leak current amplitude^29^) followed by a rapid takeoff phase. To quantify threshold temperature, we fitted a line to each of these two phases and took the intersect point of these two fitting lines as the activation threshold temperature.

### Data Analysis

Dose-response relationships were determined from normalized macroscopic current amplitudes measured at varying agonist concentrations, and fitted to the Hill equation: 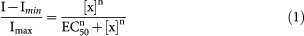


Where *I* and *I_max_* represent the steady-state currents in the presence of an agonist at concentration [*x*] and saturating concentration, respectively; *I_min_* represents the leak current or, in the case of dual-agonist experiments, the total current elicited in the presence of the first agonist; *EC_50_* represents the concentration of agonist that produces half-maximal activity; *n* is the Hill coefficient. Deactivation rate was quantified by the time it took for the current to drop from its peak level by one-half.

All values are given as mean ± SEM for the number of measurements (*n*). Statistical significance was determined using the Student's *t*-test.

## Figures and Tables

**Table 1:** Effects of extracellular NA on heat-sensing TRPV channels

	I_NA_/I_2-APB_	I_(NA + 2-APB)_/I_2-APB_	Effect on *τ*_deact_
TRPV1	-	114.5 ± 3.6% * (*n* = 3)	slow down
TRPV2	-	33.9 ± 3.5% *** (*n* = 8)	-
TRPV3	-	103.8 ± 1.2% (*n* = 4)	-
TRPV4_dm	-	32.7 ± 16.0% *** (*n* = 3)§	-

[NA] = 65 mM, [2-APB] = 1 mM.

^§^130 mM NA was used for this test.

*p < 0.05;

***p < 0.005.

**Table 2:** Effects of intracellular NA on heat-sensing TRPV channels

	I_NA_/I_2-APB_	I_(NA + 2-APB)_/I_2-APB_
TRPV1	135 ± 5% *** (*n* = 4)	120.1 ± 2.3% *** (*n* = 11)
TRPV2	-	19.0 ± 8.5% *** (*n* = 6)#
TRPV3	0.6 ± 0.1% (*n* = 5)	181.2 ± 28.4% *** (*n* = 5)§
TRPV4_dm	19.2 ± 0.1% *** (*n* = 4)	N.D.

[NA] = 65 mM, [2-APB] = 1 mM.

^#^130 mM NA and 4 mM 2-APB were used for this test.

^§^130 mM NA was used for this test.

***p < 0.005.
